# Bidirectional Mendelian randomization analysis of the causal associations between serum vitamin D levels and multiple kidney diseases

**DOI:** 10.1038/s41598-025-10305-6

**Published:** 2025-07-04

**Authors:** ShuiFang Chen, Hui Chen, XueMei Chen, Dong Zheng, YiQing Lin, YingLian Cai, Lei Yang, QianWen Zheng

**Affiliations:** 1 Department of pharmacy, Xiamen Hospital, Beijing University of Chinese Medicine, Xiamen, 361009 Fujian China; 2 Department of Nephrology, Xiamen Hospital, Beijing University of Chinese Medicine, Xiamen, 361009 Fujian China; 3 Office of Drug Clinical Trial Institutio, Xiamen Hospital, Beijing University of Chinese Medicine, Xiamen, 361009 Fujian China

**Keywords:** Genetics, Nephrology, Risk factors

## Abstract

The relationship between vitamin D levels and the risk of kidney diseases, such as IgA nephropathy (IgAN), membranous nephropathy (MN), and diabetic nephropathy (DN), is still debated in observational studies. This research aims to evaluate the causal relationships between vitamin D and these kidney diseases using a bidirectional Mendelian randomization (MR) approach. We obtained summary-level data from genome-wide association studies (GWAS) on serum 25(OH)D levels, IgAN, MN, and DN to assess the causal impact of vitamin D on these kidney diseases. The primary method used for MR analysis was the inverse variance weighted (IVW) approach. To further ascertain the stability and reliability of our results, we performed sensitivity analyses including Cochran’s Q test, MR-Egger intercept test, and leave-one-out analysis, which helped identify potential pleiotropy and outlier single nucleotide polymorphisms (SNPs) influencing the associations. Our analysis revealed no causal relationships between serum 25(OH)D levels and the risks of IgAN, MN, and DN. Sensitivity analyses confirmed the robustness of the MR findings. This MR analysis robustly refutes causal associations between genetically determined 25(OH)D levels and IgAN, MN, and DN. These null findings challenge the paradigm of vitamin D supplementation as a preventive strategy for these nephropathies, urging clinicians to prioritize interventions targeting modifiable risk factors over vitamin D optimization in kidney disease management.

Vitamin D, as a fat-soluble vitamin, plays a crucial role in maintaining various physiological processes within the human body. It is primarily known for its involvement in calcium and phosphate metabolism, which is essential for bone health^1^. Beyond its skeletal functions, vitamin D is increasingly recognized for its broader biological significance, including roles in cellular growth, neuromuscular function, and inflammation regulation^2,3^. These diverse actions highlight the importance of adequate vitamin D levels for overall health and well-being.

Recent studies indicate that vitamin D may protect against several kidney diseases, including IgA nephropathy (IgAN), membranous nephropathy (MN), and diabetic nephropathy (DN)^4,6^. The potential mechanisms through which vitamin D exerts its effects include its anti-inflammatory properties and the ability to modulate autoimmune responses. In conditions characterized by renal inflammation and fibrosis, adequate vitamin D levels may mitigate the progression of kidney damage by reducing pro-inflammatory cytokine production and enhancing immune regulation^7^. Moreover, vitamin D receptors are expressed in renal tissues, indicating a direct effect on kidney function and pathology^8^.

Emerging evidence indicates that kidney diseases may disrupt vitamin D homeostasis through multiple physiological pathways: impaired renal function reduces activity of 1α-hydroxylase, the key enzyme for vitamin D activation^9^; nephrotic syndrome leads to excessive urinary loss of vitamin D-binding protein^10^; and systemic inflammation in chronic kidney disease accelerates vitamin D catabolism^11^. These mechanisms collectively suggest that renal damage itself may actively drive vitamin D deficiency, rather than merely being its consequence.

Despite the growing body of research supporting the potential benefits of vitamin D supplementation in various kidney diseases, some studies report conflicting results. Several studies have reported that vitamin D supplementation can lead to improvements in kidney function, reduction in proteinuria, and better overall prognosis for patients with these conditions^12,13^ in contrast, other studies report no effect of vitamin D supplementation in patients with kidney disease^14^. These discrepancies highlight the need for stronger evidence to clarify the relationship between vitamin D and kidney health. A bidirectional Mendelian randomization (MR) approach offers a way to address these uncertainties. This method allows for a deeper understanding of whether vitamin D is a modifiable risk factor for improving kidney health or if its associations are merely observational^15^. Further investigation in this area could lead to more targeted therapies and improved management strategies for patients suffering from renal diseases.

MR analysis is a valuable method for studying causal relationships in epidemiology. This method, which has advanced due to the Human Genome Project, uses genetic variants as instrumental variables (IVs)^16^. This helps reduce the limitations of observational studies. As a result, MR analysis offers a clearer understanding of the causal relationships between exposures and outcomes through its unique analytical techniques. Typically, IVs are single nucleotide polymorphisms (SNPs) obtained from genome-wide association studies (GWAS). These SNPs are variations in the DNA sequence resulting from single nucleotide mutations. Therefore, this study aimed to investigate the causal relationship between serum vitamin D levels and various kidney diseases (IgAN, MN, and DN) using data from large-scale GWAS with a bidirectional MR design (Fig. 1).

## Materials and methods

**Ethics**

This research followed the STROBE-MR guidelines^17^. The data utilized in the study were sourced from publicly available databases, and thus, ethical approval was not required.

**Study design**

A bidirectional MR approach was employed to evaluate the causal relationship between vitamin D and kidney diseases. We used SNPs as IVs. These IVs helped us explore the link between vitamin D and kidney diseases. The selected SNPs satisfied the following criteria: (I) the IVs are strongly linked to the exposure (relevance); (II) the IVs are independent of any potential confounding factors (exchangeability); (III) the IVs influence the outcome solely through the exposure (exclusion restriction).

**Data sources for serum 25(OH)D levels**,** IgAN**,** MN and DN**

The summary data were obtained from Integrative Epidemiology Unit(IEU) OpenGwas and all the population in the beneath data is of European ancestry. The specifics are as follows: serum 25(OH)D levels (GWAS-ID: ebi-a-GCST90000618, sample size: 496,946, number of SNPs: 6,896,093)^18^; IgAN (GWAS-ID: ebi-a-GCST90018866, sample size: 477,784, number of SNPs: 24,182,646)^19^; MN (GWAS-ID: ebi-a-GCST010005, sample size: 7,979, number of SNPs: 5,327,688)^20^; DN (GWAS-ID: ebi-a-GCST90018832, sample size: 452,280, number of SNPs: 24,190,738)^21^.

**IVs selections**

SNPs that showed a significant association with serum 25(OH)D levels, IgAN, MN and DN were identified from GWAS data as preliminary IVs (*P* < 5 × 10^−8^ at the genome-wide significance threshold). At the same time, we conducted a linkage disequilibrium (LD) analysis to confirm the independence of the SNPs. We set the criteria as LD-r^2^ < 0.001 and a clumping distance greater than 10,000 kb. We used a weak instrumental variable (*F*-number) to examine the strength of the association between IVs and exposure, and it is generally considered that there is no weak instrument bias when *F* > 10, the calculation formula is as follows:

***R*² = 2 × EAF × (1 - EAF) ×** ***β*²**.

***F*** **= [*R*² × (*N*** **− 2)]/(1 -** ***R*²)**.

where EAF = effect allele frequency, *β* = SNP effect size, and *N* = GWAS sample size for exprosure.

Considering that the main assumption of the MR analysis is that IVs can only affect the outcome through exposure, we manually eliminated SNPs related to confounders using LDlink (https://ldlink.nih.gov/?tab=ldtrait).

**MR analysis**

We conducted bidirectional MR analyses using the TwoSampleMR R package, adhering to STROBE-MR guidelines. We used inverse variance weighting (IVW) as the primary analysis approach, and MR-Egger, the weighted median estimator (WME), weighted mode, and simple mode were used in complementary analyses^22^. IVW method estimated causal effects through a fixed-effects meta-regression model, where genetic variant-outcome associations were regressed against genetic variant-exposure associations with the intercept fixed at zero. A statistically significant association (*P* < 0.05) provided evidence for a causal relationship. Additionally, Cochran’s Q statistic was used to detect the heterogeneity. A *P*-value less than 0.05 indicates significant heterogeneity, prompting the use of a random effects model.

MR Egger accommodates pleiotropic effects for all genetic variants but conditional on the independence between pleiotropic effects and instrument strength of the variance-exposure association, with an intercept of *P* > 0.05 for non-pleiotropy. The weighted median approach provides robust estimates when ≥ 50% of instruments satisfy validity assumptions, tolerating balanced pleiotropy in remaining variants through inverse-variance weighting of SNP-outcome associations.

**Sensitivity analysis**

The leave-one-out method analyzed each SNP’s sensitivity to the outcome. This method involved removing each SNP one at a time and recalculating the effects of the remaining instrumental variables (IVs) to check if any single SNP influenced the MR estimate.

**Statistical analysis**

All analyses were performed using the TwoSampleMR package within the R environment (version 4.4.1).

## Results

**Screening of IVs**

We extracted SNPs that were strongly correlated with 25(OH)D as IVs in GWAS. Following quality control, we included 117 SNPs as IVs. Ten SNPs were included in the study when IgA was used as the exposure factor. For the IVs of MN and DN, we identified only a limited number of SNPs (*n* = 4 for MN and *n* = 1 for DN) when applying a strict *P*-value threshold (*P* < 5 × 10^−8^) for screening, so a more lenient threshold was used(*P* < 5 × 10^−6^) to include more SNPs. After eliminating SNPs related to the confounder (rheumatoid arthritis), 14 SNPs and 16 SNPs were finally obtained for the MR analysis of the causal association of MN on 25(OH)D and DN on 25(OH)D, respectively.

**25(OH)D and IgA nephropathy**

IgA nephropathy, a type of glomerulonephritis driven by immune complexes, is the most common primary glomerular disease. When serum 25(OH)D levels were used as the exposure factor, our results indicated insufficient evidence of an association between 25(OH)D and the risk of IgAN, as shown by the IVW method (*P* > 0.05) and all other MR methods (all *P* > 0.05) (Table 1). The scatter plot for MR analysis is presented in Fig. 2A. Meanwhile, Cochran’s Q test revealed no heterogeneity among SNPs (*P* > 0.05), and the MR Egger intercept test did not identify any significant horizontal pleiotropy *(P* > 0.05). The leave-one-out analysis demonstrated the outlier rs12501515, which was subsequently removed(Fig. 3A). In addition, reverse MR results revealed heterogeneity, so a random effects model was used. The results of the five methods all indicated that genetically predicted IgAN was not significantly associated with levels of serum 25(OH)D (all *P* > 0.05)(Fig. 2B). The leave-one-out sensitivity test indicated robust results (Fig. 3B).

**Table 1:** Causal association between serum 25(OH)D levels with igan, serum 25(OH)D levels with MN, and serum 25(OH)D levels with DN in MR analysis.

Exposure	Outcome	SNPs(*n*)	MR method	OR	95%CI	*P*-value
25(OH)D	IgAN	115	IVW	**0.9992**	**(0.8909**,** 1.1207)**	**0.9893**
			MR-Egger	**0.9865**	**(0.8199**,** 1.1869)**	**0.8853**
			WME	**0.9634**	**(0.8143**,** 1.1397)**	**0.6637**
			SM	**0.9451**	**(0.6418**,**1.3918)**	**0.7755**
			WM	**0.9451**	**(0.8000**,** 1.1166)**	**0.5084**
	MN	80	IVW	**1.0057**	**(0.6295**,** 1.6066)**	**0.9810**
			MR-Egger	**1.1233**	**(0.5169**,** 2.4411)**	**0.7699**
			WME	**0.7481**	**(0.3663**,** 1.5278)**	**0.4257**
			SM	**0.1598**	**(0.0354**,** 0.7218)**	**0.0195**
			WM	**0.9064**	**(0.4943**,** 1.6621)**	**0.7517**
	DN	98	IVW	**1.4074**	**(0.9447**,** 2.0968)**	**0.0929**
			MR-Egger	**1.6858**	**(0.8823**,** 3.2211)**	**0.1171**
			WME	**1.5007**	**(0.8124**,** 2.7722)**	**0.1948**
			SM	**1.2625**	**(0.3743**,** 4.2575)**	**0.7078**
			WM	**1.4540**	**(0.7962**,** 2.6551)**	**0.2261**
IgAN	25(OH)D	5	IVW	**0.9964**	**(0.9584**,** 1.0359)**	**0.8543**
			MR-Egger	**0.9089**	**(0.8516**,** 0.9700)**	**0.0636**
			WME	**0.9842**	**(0.9496**,** 1.0200)**	**0.3840**
			SM	**0.9871**	**(0.9294**,** 1.0484)**	**0.6945**
			WM	**0.9859**	**(0.9449**,** 1.0288)**	**0.5488**
MN		12	IVW	**0.9957**	**(0.9885**,** 1.0030)**	**0.2494**
			MR-Egger	**1.0118**	**(0.9799**,** 1.0448)**	**0.4890**
			WME	**1.0000**	**(0.9928**,** 1.0069)**	**0.9545**
			SM	**1.0010**	**(0.9897**,** 1.0124)**	**0.8692**
			WM	**1.0014**	**(0.9916**,** 1.0114)**	**0.7815**
DN		7	IVW	**1.0002**	**(0.9920**,** 1.0084)**	**0.9655**
			MR-Egger	**1.0102**	**(0.9992**,** 1.0213)**	**0.1293**
			WME	**1.0026**	**(0.9953**,** 1.0100)**	**0.4856**
			SM	**1.0016**	**(0.9925**,** 1.0108)**	**0.7407**
			WM	**1.0035**	**(0.9959**,** 1.0112)**	**0.4036**

**Fig. 1:**
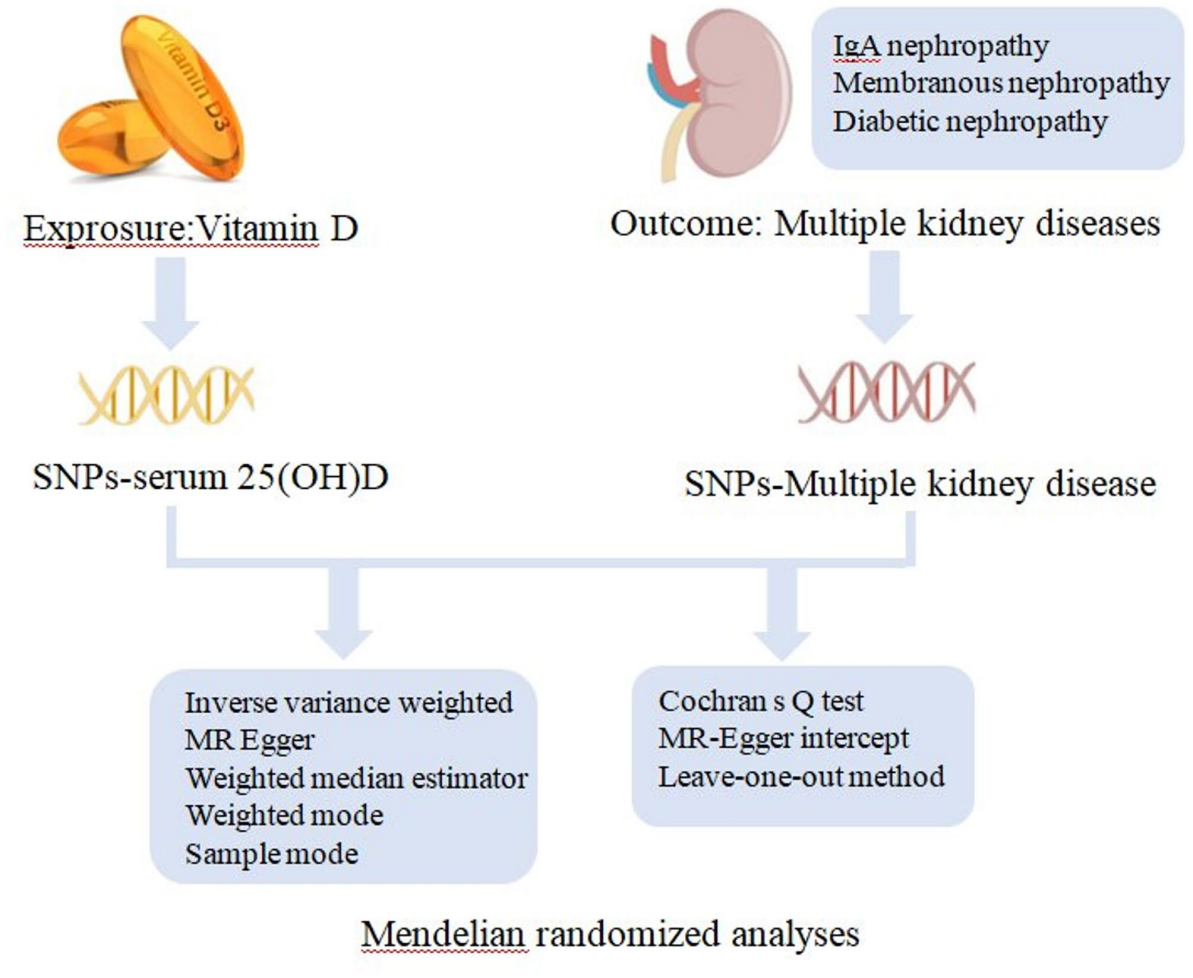
Diagram of Mendelian randomization framework in this study.

**Fig. 2:**
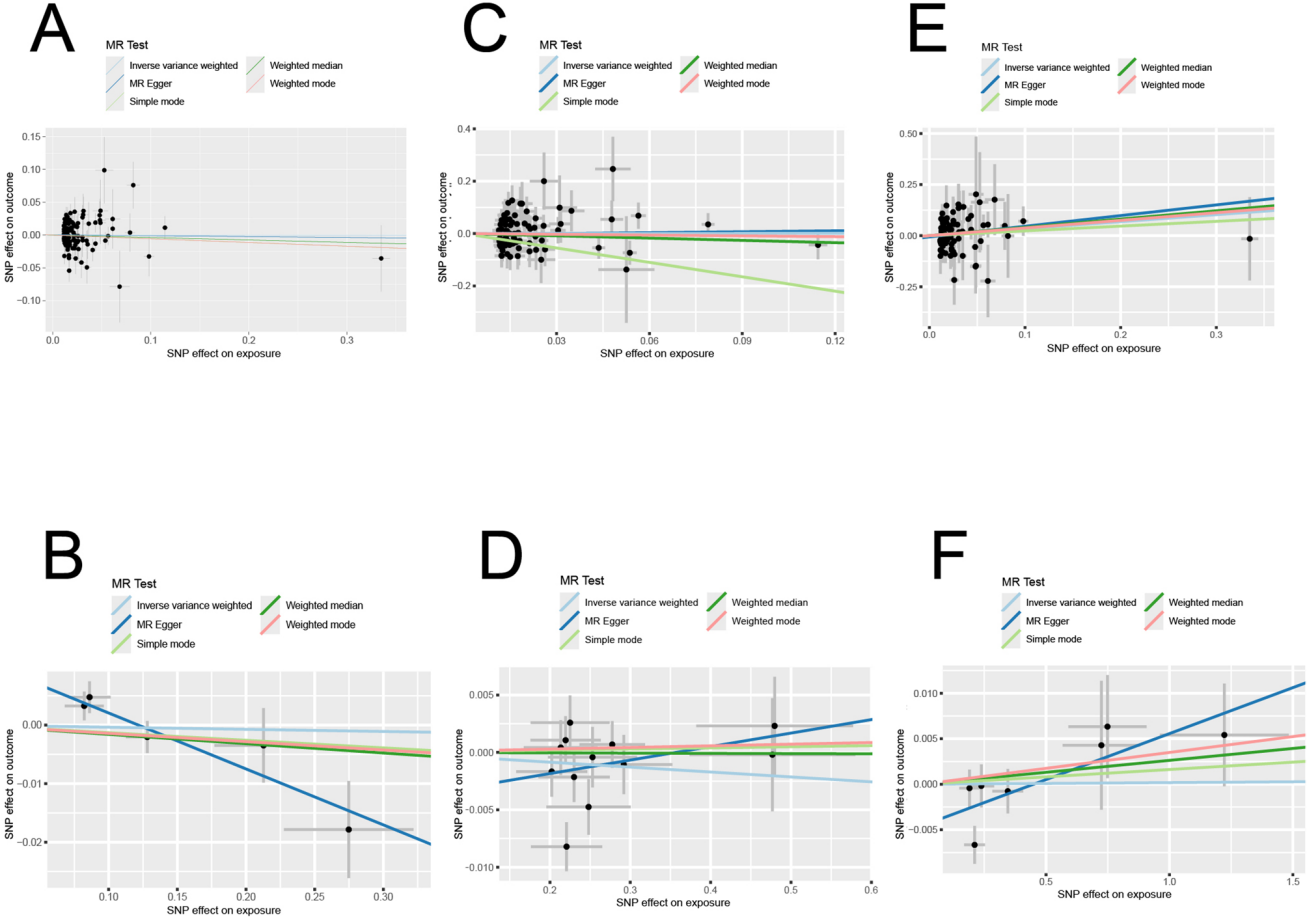
The MR analysis scatter plot for the assessment of the causal relationship between serum 25(OH)D levels and the risk of IgAN, MN and DN. Five methods including inverse variance weighting, MR-Egger, simple mode, weighted median and weighted mode were used in MR analyses. (**A**) serum 25(OH)D levels show no causal relationship with IgAN incidence (all *P* > 0.05); (**B**) IgAN show no causal relationship with serum 25(OH)D levels (all *P* > 0.05); (**C**) serum 25(OH)D levels show no causal relationship with MN incidence (all *P* > 0.05); (**D**) MN show no causal relationship with serum 25(OH)D levels (all *P* > 0.05); (**E**) serum 25(OH)D levels show no causal relationship with DN incidence (all *P* > 0.05); (**F**) DN show no causal relationship with serum 25(OH)D levels (all *P* > 0.05).

**Fig. 3:**
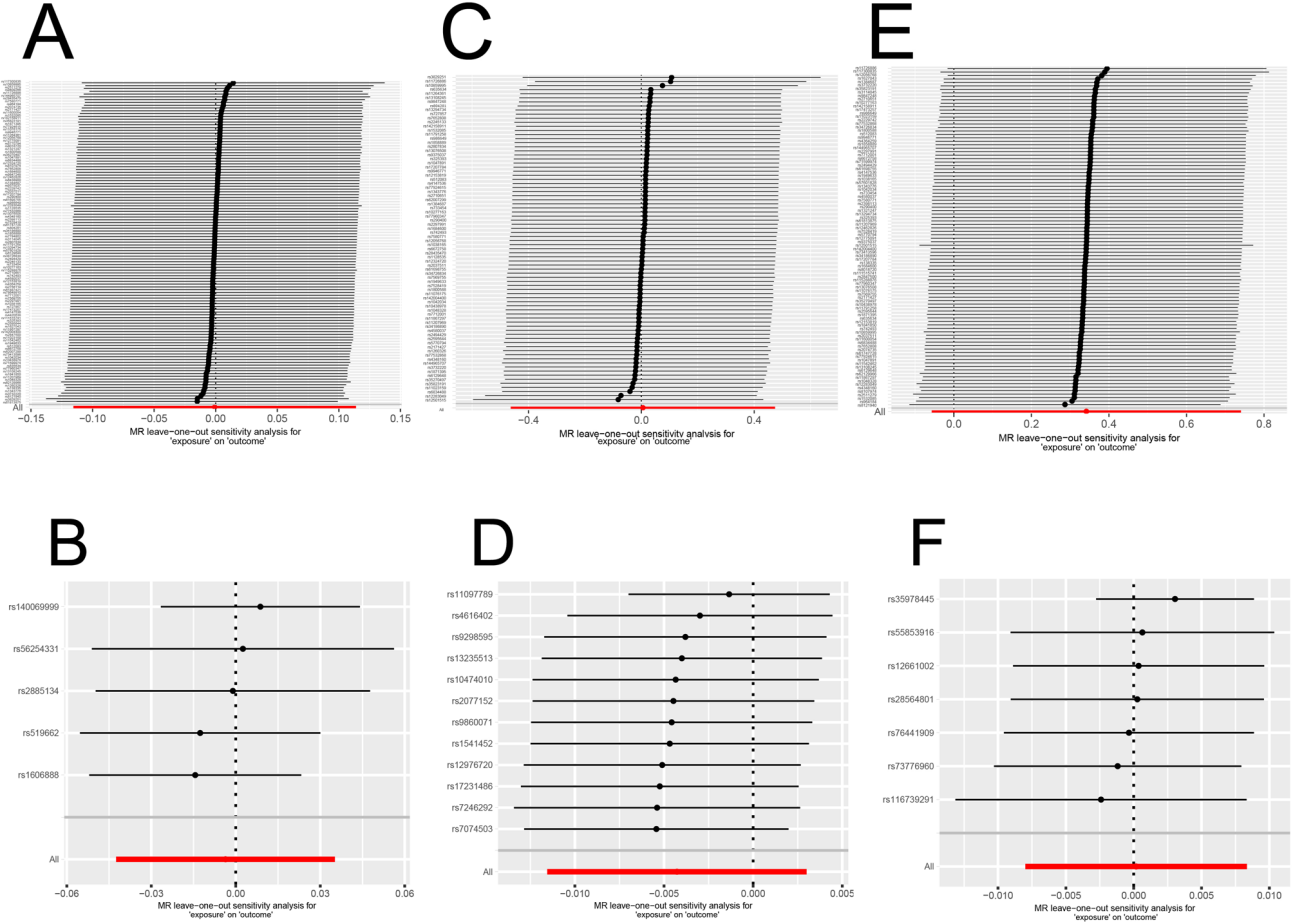
Leave-one-out sensitivity analysis of the causal association between serum 25(OH)D levels and IgAN, MN and DN (Each row represents the IVW-derived causal effect estimate after sequentially excluding the corresponding SNP; “All” indicates the causal effect estimate using all included SNPs). (**A**) serum 25(OH)D levels on IgAN, after eliminating the outlier rs12501515, the result was robust; (**B**) IgAN on serum 25(OH)D levels, no outliers were observed; (**C**) serum 25(OH)D levels on MN, no outliers were observed; (**D**) MN on serum 25(OH)D levels, no outliers were observed; (**E**) serum 25(OH)D levels on DN, after eliminating the outlier rs3829251, the result was robust; (**F**) DN on serum 25(OH)D levels, no outliers were observed.

**25(OH)D and membranous nephropathy**

MN is one of the common causes of adult-onset nephrotic syndrome, characterized by the subepithelial deposition of immune complexes on the glomerular basement membrane and diffuse thickening of the membrane. Using five MR analytical methods, we found no apparent causal relationship between 25(OH)D and the risk of MN (all *P* > 0.05). Similar results were observed in reverse MR analyses, with all *P* > 0.05 across the five methods (Table 1). The scatter plot for MR analysis is presented in Fig. 2C and D.We used a fixed-effects model when 25(OH)D was the exposure factor, as there was no heterogeneity (*P* > 0.05). In contrast, a random-effects model was employed when 25(OH)D was the outcome due to the presence of heterogeneity (*P* < 0.05). Moreover, in the MR-Egger test, there was no evidence of directional pleiotropy bias (*P* > 0.05) in either forward or reverse analysis. Additionally, the leave-one-out analysis did not reveal any SNP outliers, indicating that our results were stable (Fig. 3C and D).

**25(OH)D and diabetic nephropathy**

Diabetic nephropathy, as a major microvascular complication of diabetes, is characterized by progressive kidney dysfunction. Our study found that 25(OH)D is not associated with the risk of DN (all *P* > 0.05), and DN did not have a significant effect on serum 25(OH)D levels (all *P* > 0.05) (Table 1). The scatter plot for MR analysis is presented in Fig. 2E and F. Cochran’s Q test indicated that the SNPs of 25(OH)D showed no heterogeneity (*P* > 0.05), while those of DN displayed heterogeneity (*P* < 0.05). The MR Egger intercept test revealed no significant horizontal pleiotropy (all *P* > 0.05) in both forward and reverse analyses. Additionally, the leave-one-out analysis indicated that removing the outlier rs3829251 had no impact on the results, suggesting their robustness (Fig. 3E and F).

## Discussion

To the best of our knowledge, this study is the first bi-directional MR investigation aimed at evaluating the causal effects of vitamin D on kidney diseases, specifically IgAN, MN, and DN. However, our findings indicate no causal association between vitamin D and the three common kidney diseases as assessed by the MR approach.

Vitamin D modulates immune responses and inflammation—key pathways in IgAN pathogenesis, where immune dysregulation drives glomerular IgA deposition. While preclinical evidence indicates vitamin D mitigates renal inflammation and fibrosis^23^ and renal vitamin D receptors mediate immunomodulatory effects^24^ our bidirectional MR analysis found no causal link between vitamin D levels and IgAN risk and progression. Our results were consistent with a retrospective cohort study, including105 Indian IgA nephropathy patients demonstrated that vitamin D deficiency showed no causal association with renal outcomes^14^. This paradox may reflect the disease’s multifactorial etiology or limitations in capturing lifelong vitamin D exposure through genetic proxies. Genetic variants influencing vitamin D metabolism might also contribute to heterogeneous treatment responses observed clinically^25^. Further studies integrating longitudinal biomarkers and intervention trials are needed to clarify therapeutic potential.

Vitamin D’s potential role in MN is gaining recognition because of its ability to modulate the immune system and its importance in kidney function. MN occurs when immune complexes deposit along the glomerular basement membrane, causing damage to podocytes and resulting in proteinuria^26^. These actions suggest that sufficient vitamin D levels may protect against the development or progression of MN by reducing the inflammatory processes involved. Observational studies show a correlation between low vitamin D levels and increased severity of MN, indicating that vitamin D supplementation could provide therapeutic benefits^27^. However, our analysis using MR did not find enough evidence to prove a causal link between serum vitamin D levels and the risk or progression of MN. This finding may reflect the complexity of MN’s pathogenesis, where various factors—including genetic susceptibility, environmental triggers, and concurrent diseases—interact in ways that are not solely understood.

DN, marked by glomerular hypertrophy and extracellular matrix deposition, involves interplay of metabolic dysregulation and inflammation. Vitamin D exerts renal protection via calcium/phosphate regulation, anti-inflammatory effects, and insulin sensitization—mechanisms relevant to DN pathogenesis^28,29^. Deficiency exacerbates oxidative stress and glomerular injury^30^ yet clinical trials show conflicting results: supplementation reduces proteinuria^31^ but meta-analyses lack causal consistency^32^.

Our bi-directional MR study findings did not support a direct causal relationship between serum vitamin D levels and the risk of developing DN. Although vitamin D regulates calcium homeostasis and bone metabolism, with diverse biological roles including neuromuscular coordination, immune modulation, and regulation of cellular proliferation/differentiation, the VITAL-DKD study provides no supportive evidence for its renal protective effects in type 2 diabetes mellitus (T2DM). This 5-year prospective investigation of 1312 T2DM patients demonstrated conclusively that longitudinal variations in vitamin D levels showed no association with changes in estimated glomerular filtration rate (eGFR). Critically, routine vitamin D supplementation failed to reduce chronic kidney disease (CKD) incidence or attenuate eGFR decline in this population^33^. This lack of association may highlight the complexity of diabetic nephropathy. Multiple pathways and factors, such as glycemic control, hypertension, and genetic predisposition, interact to influence disease progression. Despite the potential benefits of vitamin D, individualized approaches to management must consider the broader context of diabetes care, including lifestyle modifications and pharmacological interventions. Future studies with larger sample sizes and robust methodologies are essential to fully elucidate the role of vitamin D in DN, potentially paving the way for novel preventive strategies in at-risk populations.

In summary, our findings highlight the complex relationship between vitamin D levels and various kidney diseases. While we didn’t observe significant associations between vitamin D and these renal conditions, it is essential to consider the broader context of vitamin D’s role in immune system regulation. Multiple MR studies have examined the potential link between vitamin D and various immune-mediated diseases, suggesting that vitamin D may not have a causal effect on many immune disorders^34,35^. This discrepancy raises intriguing questions about the mechanisms through which vitamin D influences kidney health and its potential pathways of action within the immune system. while circulating 25(OH)D serves as the conventional biomarker, renal 1α-hydroxylase activity determines local 1,25(OH)₂D₃ levels-a critical distinction highlighted by the ViRTUE-CKD tria^36^ where vitamin D receptor activator paricalcitol supplementation failed to alter kidney outcomes.

In this study, we rigorously addressed pleiotropy through: (I) MR-Egger regression showing no horizontal pleiotropy (intercept *P* > 0.05 for all exposures); (II) LDlink-based pruning(r²<0.001) to exclude SNPs correlated with confounding traits; (III)F-statistics > 10 confirming strong instruments; (IV) Cochran’s Q tests demonstrated minimal heterogeneity (*P* = 0.0544–0.7066), supporting effect homogeneity and the leave-one-out method was used to eliminate outliers(rs3829251/rs12501515).

This study’s strength lies in its large-scale MR analysis, which utilized multiple GWAS datasets to systematically evaluate the causal relationship between serum 25(OH)D levels and the risk of various kidney diseases. However, MR has its limitations. One potential drawback is the reliance on genetic variants that might not fully capture the biological pathways through which vitamin D affects health. Additionally, gene-environment interactions may complicate interpretations. Certain genetic variants might affect how environmental factors, like vitamin D from sunlight or diet, interact with kidney disease risk. Furthermore, since the study focused on a population of European ancestry, caution is warranted when generalizing the findings to other populations.

The application of relaxed instrument selection criteria (*P* < 5 × 10⁻⁶) for MN and DN analyses was necessitated by insufficient SNP availability at conventional genome-wide significance (*P* < 5 × 10⁻⁸), yielding only 4 SNPs for MN and 1 SNP for DN. This threshold adjustment increased instrument counts to 14 SNPs for MN and 16 SNPs for DN while maintaining adequate instrument strength (all *F* > 10). To address potential weak instrument bias, our sensitivity framework incorporated MR-Egger intercept tests (MN: *P* = 0.34; DN: *P* = 0.08), and leave-one-out analysis confirming effect stability. The limited sample size for MN represents a critical constraint in our study. We fully acknowledge this important issue related to sample size limitations. To address this, future research will involve increasing the MN sample size and conducting cross-ethnic meta-analyses. Additionally, we plan to incorporate multi-omics integration studies, which will enhance the reliability of our findings. By expanding our participant base and employing diverse methodologies, we aim to better elucidate the complex relationship between serum vitamin D levels and kidney diseases.

## Conclusions

In conclusion, this large-scale bidirectional MR study provides evidence refuting causal relationships between serum 25(OH)D levels and IgAN, MN, or DN. These findings challenge empirical clinical practices that empirically prescribe vitamin D for renal protection in these diseases, particularly among populations without severe deficiency. Clinicians should prioritize established interventions—such as RAAS inhibition in IgAN, control of blood sugar in DN and immunosuppression optimization in MN—over vitamin D status monitoring. While vitamin D analogs retain therapeutic value in managing chronic kidney disease-mineral bone disorders (CKD-MBD), their preventive application in early-stage nephropathies lacks biological justification. In summary, Our MR study’s lack of convincing evidence linking vitamin D to kidney disease risk underscores a crucial public health message.

## Data Availability

The datasets examined in this study can be accessed through the IEU Open GWAS Project.
